# Gd/Sm-Pr Co-Doped Ceria: A First Report of the Precipitation Method Effect on Flash Sintering

**DOI:** 10.3390/ma12081218

**Published:** 2019-04-14

**Authors:** Luca Spiridigliozzi, Lorenzo Pinter, Mattia Biesuz, Gianfranco Dell’Agli, Grazia Accardo, Vincenzo M. Sglavo

**Affiliations:** 1 Department of Civil and Mechanical Engineering, University of Cassino and Southern Lazio, Via G. Di Biasio 43, 03043 Cassino (FR), Italy; l.spiridigliozzi@unicas.it; 2 Department of Industrial Engineering, University of Trento, Via Sommarive 9, 38123 Trento, Italy; lorenzo.pinter@alumni.unitn.it (L.P.); mattia.biesuz@unitn.it (M.B.); vincenzo.sglavo@unitn.it (V.M.S.); 3 Center of Hydrogen-Fuel Cell Research, Korea Institute of Science and Technology, Hwarangno 14-gil, Seongbuk-gu, Seoul 136-791, Korea; d16605@kist.re.kr

**Keywords:** co-doped ceria, flash sintering, precipitation

## Abstract

In this work, ceria-based ceramics with the composition Gd_0.14_Pr_0.06_Ce_0.8_O_2-δ_ and Sm_0.14_Pr_0.06_Ce_0.8_O_2-δ_, were synthesized by a simple co-precipitation process using either ammonium carbonate or ammonia solution as a precipitating agent. After the calcination, all of the produced samples were constituted by fluorite-structured ceria only, thus showing that both dopant and co-dopant cations were dissolved in the fluorite lattice. The ceria-based nanopowders were uniaxially compacted and consequently flash-sintered using different electrical cycles (including current-ramps). Different results were obtained as a function of both the adopted precipitating agent and the applied electrical cycle. In particular, highly densified products were obtained using current-ramps instead of “traditional” flash treatments (with the power source switching from voltage to current control at the flash event). Moreover, the powders that were synthesized using ammonia solution exhibited a low tendency to hotspot formation, whereas the materials obtained using carbonates as the precipitating agent were highly inhomogeneous. This points out for the first time the unexpected relevance of the precipitating agent (and of the powder shape/degree of agglomeration) for the flash sintering behavior.

## 1. Introduction

One of the main targets of solid oxide fuel cell (SOFC) research regards decreasing their operative temperature to 500–700 °C, towards the future generation of so-called intermediate temperature SOFC. With this in mind, it is necessary to use ceramic electrolytes with both chemical/mechanical stability and adequately high ionic conductivity in said temperature range [1]. Ceria (CeO_2_)-based ceramics are considered to be promising candidates, especially when doped with aliovalent cations (Gd or Sm) or co-doped with other rare-earth elements (i.e., Pr, Er, Nd) or earth-alkaline elements (i.e., Ca, Sr). These allow for an increase in the overall concentration of oxygen vacancies and a tuning of their final electrochemical properties [2,3,4,5]. Recently, a certain interest has been generated by the very positive effect of Pr-co-doping in terms of electric properties and sintering aptitude [6,7].

Nevertheless, ceria-based materials generally exhibit poor sinterability, meaning that high temperatures and long timeframes are necessary to achieve adequate densification [8]. To overcome this major drawback, many strategies have been proposed. These strategies include the use of more reactive ceria powders synthesized by co-precipitation [9,10,11], the sol–gel method [12], and hydrothermal treatments [13,14], or the improvement of the sintering cycle by using sintering aids [15], or using innovative densification techniques [16,17]. Among these methods, flash sintering (FS) has recently been shown to be a very promising consolidation route suitable for densifying ceramics at a reduced temperature via the application of an external electric field [18,19,20]. When a critical combination of electric field and temperature is reached, a power surge [21,22] occurs (the so-called flash event), which is associated with a nearly instantaneous densification. Since FS is usually carried out by voltage-limited and current-limited power sources, and upon the rapid electrical conductivity increase, three stages can be identified throughout the process: stage I (or incubation), where the power source works in voltage control; stage II (or flash event), where the current abnormally increases and reaches the limit of the system; and stage III, where the system is current-limited. One of the main drawbacks of FS is the formation of preferential current paths, also known as hotspots [23], that can be limited by avoiding the power spike of the flash transition through a progressive and constant intensification of the imposed current [24,25,26].

Although several reports can be found in the literature concerning the application of FS to doped-ceria materials [1,27,28,29,30], no studies concerning FS applied to ceria doped by metals with RedOx behavior have been reported. In the present work, FS was applied to Gd/Pr and Sm/Pr co-doped ceria. Both materials were synthesized via a co-precipitation route using ammonium carbonate or ammonia solution as a precipitating agent, and the relevant effect on the field-assisted sintering process was analyzed in detail.

## 2. Materials and Methods 

Cerium(III) nitrate (Ce(NO_3_)_3_·6H_2_O, 99.0%, Sigma-Aldrich, Milan, Italy), Samarium(III) nitrate (Sm(NO_3_)_3_·6H_2_O, 99.9%, Sigma-Aldrich, Milan, Italy), Gadolinium(III) nitrate (Gd(NO_3_)_3_·6H_2_O, 99.0% pure, Sigma-Aldrich, Milan, Italy), and Praseodymium(III) nitrate (Pr(NO_3_)_3_·6H_2_O, 99.0%, Sigma-Aldrich, Milan, Italy) were used as metal precursors for the syntheses of the various samples. Ammonia solution (30 wt%, Carlo Erba, Cornaredo, Italy) and ammonium carbonate ((NH_4_)_2_CO_3_, 99.0%, Fluka-Honeywell, Sigma-Aldrich, Steinheim, Germany) were used as precipitating agents. 

In a typical synthesis, the proper amount of rare-earth nitrates was initially dissolved in de-ionized water to reach a total cationic concentration of 0.1 M (solution A). Then, an aqueous solution containing 0.5 M ammonium carbonate (solution B) or a solution containing about 2 M NH_3_ (solution C) was prepared as a precipitating agent. The co-precipitation was carried out at room temperature by quickly adding solution B to solution A, or solution C to solution A, all kept in vigorous agitation, and by using a slight excess (~ 50%) of the added base. When the two solutions were mixed, the co-precipitate instantly formed. It was then recovered by vacuum filtration, repeatedly washed with de-ionized water, and finally dried overnight at 80 °C.

Three different powders were prepared: Gd_0.14_Pr_0.06_Ce_0.8_O_2-δ_ with ammonium carbonate (GPDC-AC), Sm_0.14_Pr_0.06_Ce_0.8_O_2-δ_ with ammonium carbonate (SPDC-AC), and Sm_0.14_Pr_0.06_Ce_0.8_O_2-δ_ with ammonia solution (SPDC-NH3).

The synthesized powders were calcined at 550 °C for 30 min under static air to induce the thermal decomposition and the complete crystallization in the fluorite structure of ceria-based compounds.

All synthesized powders were characterized by X-ray powder diffraction (XRD) by using an X’PERT diffractometer (Panalytical, Almelo, The Netherlands) with CuKα radiation.

The thermal behavior of the powders was analyzed by differential thermal analysis and thermogravimetric analysis (DTA-TG) either in air or in Ar (STA 409 Thermoanalyzer, Netzsch, Selb, Germany), with a heating rate of 10 °C/min up to 1200 °C and α-Al_2_O_3_ as a reference.

The calcined powders were subsequently used to produce cylindrical pellets (about 4 mm thick, 8 mm in diameter) by uniaxial pressing (200 MPa) which were flash-sintered in air under different electrical conditions. 

The powder morphology and sintered pellet microstructure was studied by SEM (Novasem, FEI Co., Hillsboro, OR, USA) equipped with a standard Everhart-Thornley detector (ETD) and a through lens detector (TLD) to improve the observation of crystal surfaces at higher magnifications.

Flash sintering experiments were carried out within a modified Linseis L75 dilatometer using a heating rate of 20 °C/min, from room temperature and up to 10 °C above the registered flash-onset temperature (about 750–800 °C), with electric fields of 50 V/cm and current limits between 20 and 80 mA/mm^2^. The flat surfaces of the pellets were painted using silver paste and the samples were then introduced into the dilatometer between two Pt disks used as electrodes. The electric power was provided by a DC power source Sorensen DLM300 (Sorensen, San Diego, CA, USA). Once the power source switched from voltage to current control the sample entered into the so-called flash state, after which the current was left to flow for 30 s. Then, both the power source and the furnace were switched off. The electrical data were controlled by a Keithley 2000 multimeter with an acquisition frequency of 1 Hz.

In addition to “conventional” flash experiments, flash sintering using a current-ramp approach was also employed. In this case, treatments were carried out at a constant furnace temperature of 700 °C. Two different ramps were used. In the first one, the current was increase by 2 mA/mm^2^ steps every 30 s up to 12 mA/mm^2^, while in the second one, the current was increase by 5 mA/mm^2^ steps every 30 s up to 20 mA/mm^2^.

## 3. Results and Discussion

### 3.1. Characterization of the Samples

**Figure 1:**
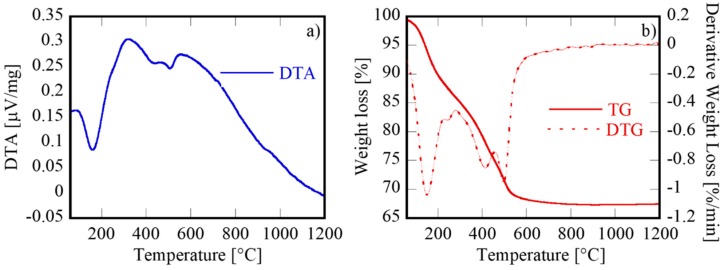
DTA (**a**)-TG/DTG (**b**) of GPDC-AC.

Through the described co-precipitation process in the presence of ammonium carbonate as a precipitating agent, cerium-based co-precipitates are always amorphous in nature, regardless of cerium doping, as clearly stated in our previous works [7,9]. According to these, the as-formed amorphous phase is very likely constituted by a rare-earth (RE) hydrate hydroxide carbonate (RECO_3_OH·xH_2_O, where in the present work RE = Pr_0.06_Gd_0.14_Ce_0.80_, or RE = Pr_0.06_Sm_0.14_Ce_0.80_). This was confirmed by DTA-TG carried out in Ar on a GPDC-AC sample, as shown in Figure 1. The total weight loss (32.5 wt%) was divided into three well-distinct events, clearly visible from the TG derivative curve (DTG), where three points with a maximum weight loss rate located at about 160 °C, 400 °C, and 500 °C were identified. This behavior agreed very well with the decomposition process of RECO_3_OH·xH_2_O, which can be represented by the following chemical reactions [31]:

(1) $$2{\mathrm{REOHCO}}_{3}\cdot {\mathrm{xH}}_{2}O\to {\;\mathrm{RE}}_{2}O{\left({\mathrm{CO}}_{3}\right)}_{2}+\left(2x+1\right)H_{2}O↑$$

(2) $${\mathrm{RE}}_{2}O{\left({\mathrm{CO}}_{3}\right)}_{2}\to {\;\mathrm{RE}}_{2}O_{2}{\mathrm{CO}}_{3}+{\mathrm{CO}}_{2}↑$$

(3) $${\mathrm{RE}}_{2}O_{2}{\mathrm{CO}}_{3}\to {\;\mathrm{RE}}_{2}O_{3}+{\mathrm{CO}}_{2}↑$$

This mechanism applies in air when RE is a rare-earth with a fixed 3+ valency. In the case of Ce^3+^, which can very easily oxidize to Ce^4+^ in air, thermal treatment in a non-oxidizing atmosphere (such as the Ar-based atmosphere used for DTA-TG) is needed to highlight this three-step behavior. In our case, we considered RE to be a mixture of Ce, Gd, and Pr according to the initial composition (i.e., RE = Gd_0.14_Pr_0.06_Ce_0.80_). Therefore, reactions (1), (2), and (3) corresponded to the peaks at 160 °C, 400 °C, and 500 °C, respectively. The theoretical total weight loss depended on the x-value, and when x = 1.5, it was equal to 32.5%, i.e., in perfect agreement with the measured value. Therefore, it was reasonable to assume that the composition of the amorphous co-precipitate corresponded to (Gd_0.14_Pr_0.06_Ce_0.80_)CO_3_OH·1.5H_2_O.

All thermal events occurred well below 600 °C. Therefore, the as-obtained samples were calcined (under static air) at 550 °C for 30 min to decompose the hydroxycarbonate, oxidize Ce^3+^ to Ce^4+^, and induce the crystallization of the fluorite phase. Such mild conditions for the calcination step were selected in order to limit undesired phenomena, such as excessive (and unnecessary) grain growth and a consequent reduction of the powders’ reactivity.

**Figure 2:**
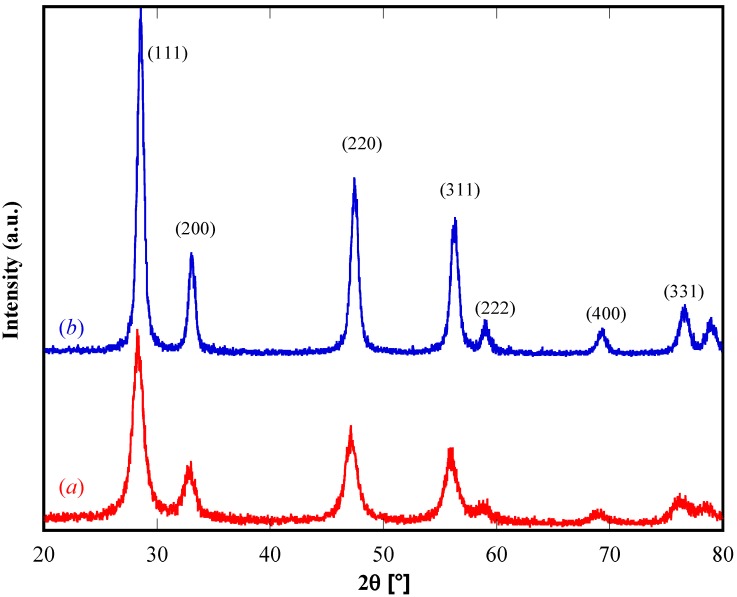
XRD patterns of as-synthesized (red) and calcined (blue) SPDC-NH3.

The SPDC-NH3 exhibited very different behavior. It was already partially crystallized in the fluorite-like phase after co-precipitation, as was evident from the diffraction pattern shown in Figure 2, where only fluorite-structured ceria peaks appeared, corresponding to a very fine nanometric structure that grew slightly after calcination at 550 °C. 

Thus, the presence of Pr as a co-dopant does not influence the co-precipitation behavior, as has already been reported in the literature for pure and single metal-doped ceria produced using ammonium carbonate [32,33] and ammonia solution [34]. The diffraction patterns of the other calcined samples were nearly identical to those in Figure 2, thus confirming a fluorite structure as for the ICDD card n. 75-158 (corresponding to Sm_0.2_Ce_0.8_O_1.90_). Nevertheless, a very slight shift of the peaks position was identified with respect to the reference card, and this confirmed that the Pr forms a stable, solid solution with ceria in the considered specimens. The cell parameter of calcined SPDC-NH_3_, calculated using MAUD [35], was 0.5430(1) nm. The theoretical cell parameter reported in the ICDD card n. 75-158 was 0.5433 nm. Our sample could be considered as the reference sample (Sm_0.20_Ce_0.80_O_1.90_), where 0.06 Sm^3+^ ions are replaced by 0.06 Pr^3+^ ions. Now, considering that the ionic radii in eightfold coordination for Sm^3+^ and Pr^3+^ were 0.1079 nm and 0.1126 nm, respectively [36], the cell parameter *a* (in nm) of the fluorite-structured ceria at room temperature was calculated according to Vegard’s law [37]:

(4) $$a=0.5413+\underset{i}{\sum }\left(0.022\Delta r_{i}+0.00015\Delta z_{i}\right)m_{i}$$

where Δr_i_ is the difference between the ionic radius of the dopant ions (i.e., Sm and Pr) and Ce^4+^ in eightfold coordination, Δz_i_ is the charge difference between the dopants and Ce^4+^, and *m_i_* is the concentration (mol%) of the i-th dopant expressed as MOx. The calculated cell parameter was 0.5437 nm. The small but not negligible difference compared to the calculated value from the diffraction data was very likely correlated with the partial oxidation of Pr^3+^ to Pr^4+^ according to the following reaction:

(5) $$2{\mathrm{Pr}}_{\mathrm{Ce}}^{\prime }+V_{O}^{..}+\frac{1}{2}O_{2}\left(g\right)↔2{\mathrm{Pr}}_{\mathrm{Ce}}^{X}+O_{O}^{X}$$

Consequently, the cell parameter varied and, in particular, decreased, as the ionic radius of Pr^4+^ was equal to 0.096 nm. In addition, using the mixture rule and calculating the theoretical cell parameter using Equation (4) in the extreme cases where Pr was completely 3+ or 4+, it was possible to estimate the fraction of Pr^3+^ with respect to the whole Pr amount. This value was equal to 0.45 and, therefore, about one-half of the Pr^3+^ cations were oxidized to Pr^4+^ after calcination. On this basis, the composition of the ceria-based samples after calcination was Sm_0.14_Pr(III)_0.027_Pr(IV)_0.033_O_2-δ_. In addition, during any thermal treatment, the RedOx behavior represented by Equation (5) was able to modify the ratio Pr^3+^/Pr^4+^ with important consequences for its electrical and transport properties, as pointed out in previous works [7,9], where Pr was shown to improve both sintering aptitude and electrical conductivity.

**Figure 3:**
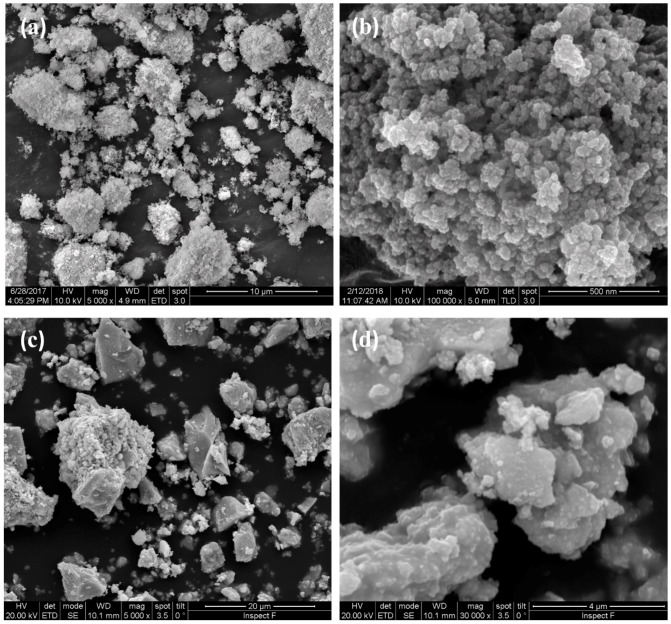
SEM micrographs of calcined SPDC-AC (**a**,**b**) and SPDC-NH3 (**c**,**d**) powders.

Figure 3 shows some SEM micrographs of SPDC-AC and SPDC-NH3 calcined powders. The morphology of the samples appeared to be clearly different. SPDC-AC specimens were formed by isometric granules constituted by very small (around a few tenths of nanometers in diameter) spherical particles. This revealed that the calcination step induced negligible grain growth or coarsening. Conversely, SPDC-NH3 powder was constituted by irregular-shaped particles with very variable sizes, from less than a micron to about 10 µm.

### 3.2. Flash Sintering

For the field-assisted sintering runs, several pellets were produced by uniaxial pressing at 200 MPa. The green density of the SPDC-AC and GPDC-AC samples ranged between 40%–45%, whilst a slightly higher green density (around 50%) was measured for SPDC-NH3. We believe that this small difference in green density had negligible consequences on the sintering behavior of the samples.

**Figure 4:**
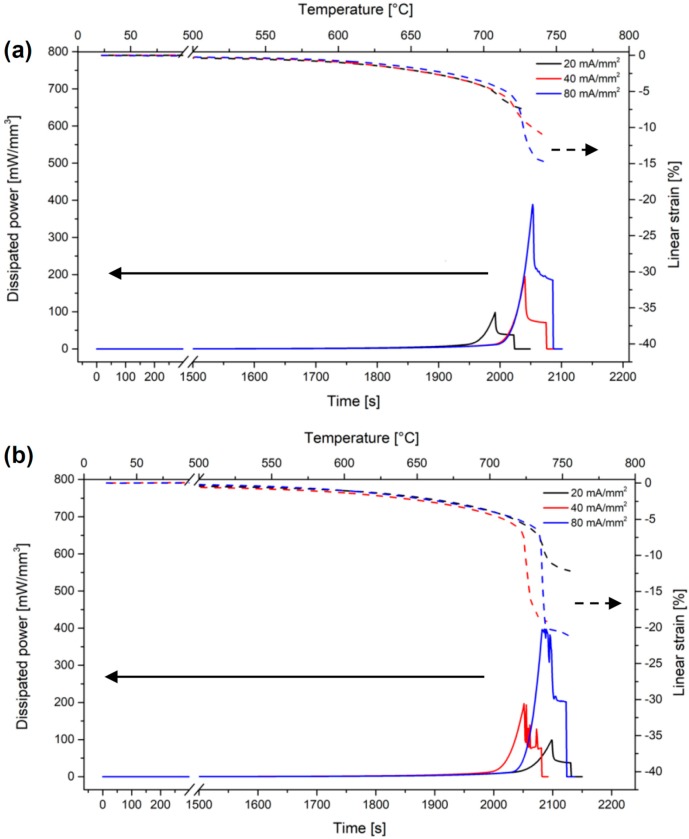
Specific power density and deformation for SPDC-AC (**a**) and GPDC-AC (**b**) samples sintered under 50 V/cm and with different current limits.

Several tests were carried out on SPDC-AC and GPDC-AC pellets, using an electric field of 50 V/cm and different current limits (Figure 4). In all cases, the onset of the flash transition was between 700 and 750 °C for both compositions, as testified by the presence of a peak in the specific power dissipation. The onset temperature was substantially aligned with those measured in previous works on variously doped ceria-based ceramics [27,28]. Nevertheless, the final relative density was rather low, ranging between 70% and 90%. In fact, even though to the best of our knowledge no data on density after flash sintering is available for materials with the composition studied in this work, higher densities (in the range 93–99 %) were obtained for ceria-based materials with different compositions (i.e., not including Pr) [27,28]. The samples treated with the largest current (80 mA/mm^2^) appeared broken after the flash, this pointed out an inhomogeneous response to the flash treatment with the development of microstructural/thermal gradients and stresses.

**Figure 5:**
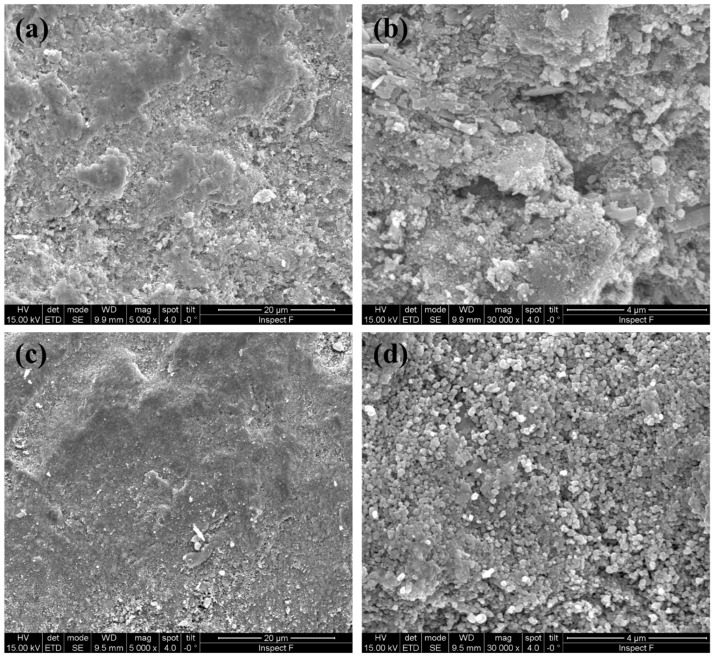
SEM micrographs of flash-sintered SPDC-AC (**a**,**b**) and GPDC-AC (**c**,**d**) samples under a current limit of 20 mA/mm^2^.

The morphology of the flash-sintered SPDC-AC and GPDC-AC pellets is shown in Figure 5. The micrographs confirm the rather low densification, especially for the former material, the density of which was around 80%. This also showed a heterogeneous microstructure. Heterogeneities in flash-sintered components are well known and are usually due to the formation of preferential paths where the electrical current concentrates. The measured densities are reported in Table 1. We observed a quite large scatter in the reported values, which correlated with the formation of non-controllable and non-reproducible current paths, which originated from casual temperature fluctuation or uncontrollable effects on the electrodes or small heterogeneities in green density.

**Figure 6:**
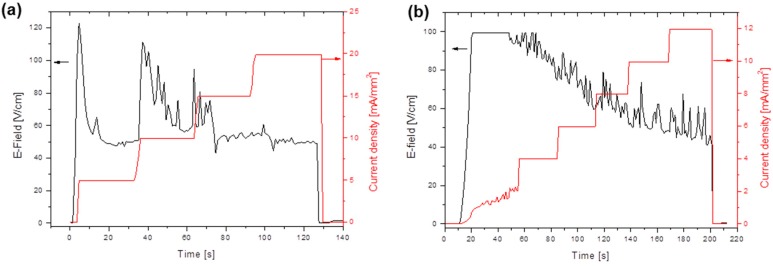
Electrical data for the two current ramps adopted in this work: up to 20 mA/mm^2^ (**a**) and 12 mA/mm^2^ (**b**).

In order to reduce the inhomogeneity and improve the densification, current ramp flash sintering was carried out by increasing the current limit by 2 mA/mm^2^ every 30 s up to a maximum of 12 mA/mm^2^, and by 5 mA/mm^2^ steps up to 20 mA/mm^2^ (Figure 6). Dong [23] has shown that the hotspots likely form during the flash transition and are more stable at higher electric power peaks. Therefore, the absence of the power peak, which is typical of the flash transition in current-ramp experiments, is expected to reduce the formation of preferential current paths. 

**Figure 7:**
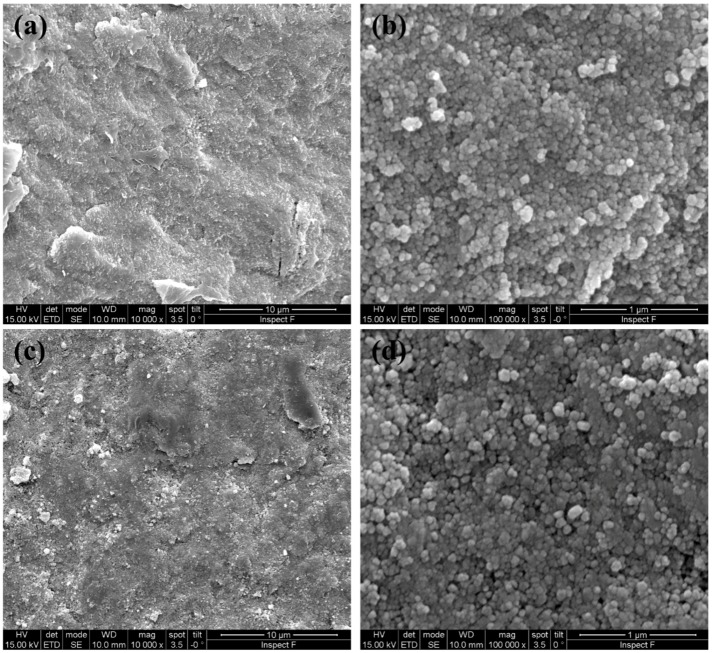
SEM micrographs of sintered SPDC-AC with ramp cycle; ramp up to 12 mA/mm^2^ (**a**,**b**) and ramp up to 18 mA/mm^2^ (**c**,**d**).

In the present work, current ramp flash sintering was very effective both for SPDC-AC and GPDC-AC powders, and relative densities of around 90% or even larger were obtained. The density measurements were confirmed by the SEM micrographs shown in Figure 7 (for the case of SPDC-AC), which showed well-densified regions. The material had a homogeneous microstructure with an extremely limited grain size (only some tenths of nanometers). Such a fine-grained microstructure appeared surprising considering the rather “long” treating times used during the current ramps, pointing out a reduction of the grain coarsening phenomena during field-assisted sintering. We can therefore state that SPDC-AC and GPDC-AC can only be successfully flash-sintered using the current ramp approach, whereas “traditional” flash sintering is not effective.

At this point, we questioned the origin of the limited densification attained by “traditional” flash sintering in the SPDC-AC and GPDC-AC samples. This can be a result of the composition (i.e., ceria doped with elements with RedOx behavior, like Pr, which has not been tested before), or it can be related to the precipitation process (i.e., with the powder morphological characteristics shown before). To answer this question, we carried out some “traditional” flash sintering experiments on GDC and SDC powder (i.e., without Pr in the fluorite lattice) synthesized using ammonium carbonate as a precipitating agent. The sintered samples were characterized by a porous microstructure after the “traditional” flash, which suggests that Pr doping was not the real origin of the inhomogeneity and poor densification.

**Figure 8:**
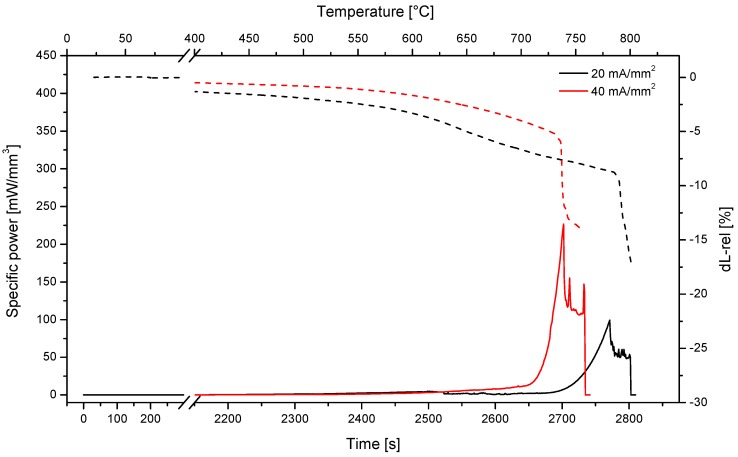
Specific power density and dilatometric plot for SPDC-NH3 samples treated using 50 V/cm and different current limits.

**Figure 9:**
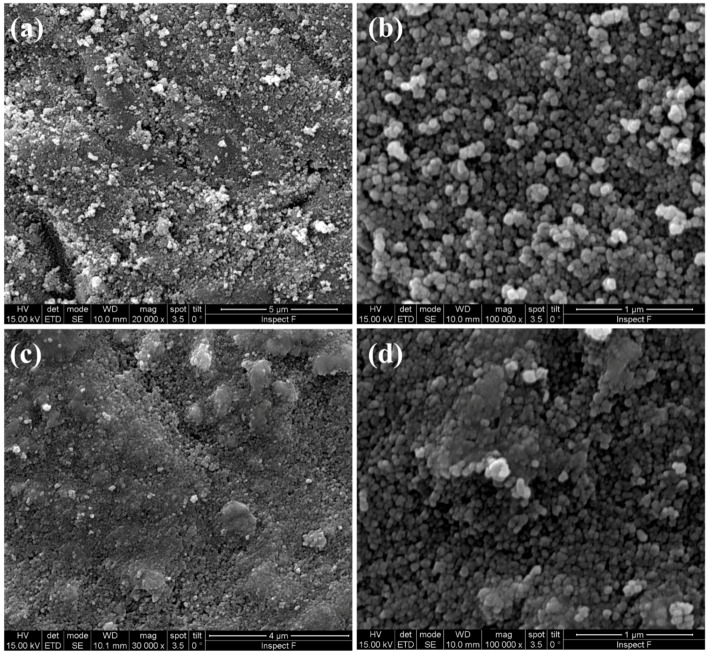
SEM micrographs of sintered SPDC-NH3 at 50 V/cm and 20 mA/mm^2^ (**a**,**b**) and 40 mA/mm^2^ (**c**,**d**).

Additional experiments were carried out on the SPDC-NH3 sample. The power dissipation during the process and the dilatometric plots are shown in Figure 8, which highlights the presence of the flash transition at 730–760 °C. The relative density of these samples was around 93% for both. The microstructure shown in Figure 9 appears to be rather well-densified. The samples synthesized using ammonia solution as a precipitating agent were effectively densified by “traditional” flash sintering cycles. A summary of all of the samples produced in this work with the obtained densities is reported in Table 1.

The results suggest that there was a strong effect of the used precipitating agent on the final microstructure of the flash sintering sample, and that the used precipitating agent can impact significantly on the homogeneity of the obtained materials. The origin of this effect still appears not to be completely clear. The morphological differences between the calcined powders, as pointed out in Figure 3, could play an important role in this regard. We can also specify that SPDC-AC and SPDC-NH3 behave differently in the flash state. In fact, while for SPDC-AC the power dissipation after the flash transition was 41 ± 5 and 78 ± 15 mW/mm^3^ for current limits of 20 and 40 mA/mm^2^, respectively, the power increased to 55 ± 6 and 119 ± 14 mW/mm^3^ in the case of SPDC-NH3 (for the same currents). This suggests that the SPDC-AC samples became more conductive than SPDC-NH3 as they sintered upon flash. The decrease of the electrical resistivity during the flash transition is a possible origin of inhomogeneity, any casual positive perturbation in the sample temperature would increase the local conductivity, making the hotspot stable. Conversely, if the conductivity increases less sharply during the flash, hotspots are less likely to be formed.

A deep understanding of the relation between the precipitating agent, morphology of powder/agglomerates, and flash sintering behavior deserves additional future investigation.

## 4. Conclusions

In this work, we investigated the flash sintering behavior of Gd/Sm-Pr co-doped ceria produced by co-precipitation. The results indicate that there was a strong effect of the precipitating agent on the flash sintering behavior, where the materials produced with NH_3_ as a precipitating agent were much denser and more homogeneous than those co-precipitated using ammonium carbonate. The origin of this difference is still not completely understood, but a key effect of the morphological properties of the powder on the flash process seems to have been suggested.

We also showed that the powder that cannot be densified by “traditional” flash experiments (i.e., with the switch from voltage to current control), can however be sintered using a flash current-ramp. Current ramp flash sintering appears therefore to be a powerful tool for improving the homogeneity of flash-sintered components. 

## Figures and Tables

**Table 1:** Summary of the different treating conditions and final densities achieved in this work.

Composition	Precipitating Agent	Flash Cycle	Electric Data	Relative Density [%]
6PrSDC20	(NH_4_)_2_CO_3_	Traditional	50V/cm, 20 mA/mm^2^	81
6PrSDC20	(NH_4_)_2_CO_3_	Traditional	50V/cm, 40 mA/mm^2^	61
6PrSDC20	(NH_4_)_2_CO_3_	Traditional	50V/cm, 80 mA/mm^2^	74
6PrGDC20	(NH_4_)_2_CO_3_	Traditional	50V/cm, 20 mA/mm^2^	88
6PrGDC20	(NH_4_)_2_CO_3_	Traditional	50V/cm, 40 mA/mm^2^	77
6PrGDC20	(NH_4_)_2_CO_3_	Traditional	50V/cm, 80 mA/mm^2^	60
GDC20	(NH_4_)_2_CO_3_	Traditional	50V/cm, 15 mA/mm^2^	66
GDC20	(NH_4_)_2_CO_3_	Traditional	50V/cm, 10 mA/mm^2^	68
SDC20	(NH_4_)_2_CO_3_	Traditional	50V/cm, 15 mA/mm^2^	84
6PrSDC20	(NH_4_)_2_CO_3_	Ramp	5 mA/mm^2^ steps up to 20 mA/mm^2^	91
6PrSDC20	(NH_4_)_2_CO_3_	Ramp	2 mA/mm^2^ steps up to 12 mA/mm^2^	88
6PrGDC20	(NH_4_)_2_CO_3_	Ramp	2 mA/mm^2^ steps up to 12 mA/mm^2^	93
6PrSDC20	NH_3_	Traditional	50V/cm, 20 mA/mm^2^	94
6PrSDC20	NH_3_	Traditional	50V/cm, 40 mA/mm^2^	93
